# Supplementary material for: Ultraviolet Photodetector Based on a Beta-Gallium Oxide/Nickel Oxide/Beta-Gallium Oxide Heterojunction Structure
Source: Sensors (Basel). 2023 Oct 9;23(19):8332. doi: 10.3390/s23198332 (PMC10575374; doi:10.3390/s23198332)
Supplement: Supplementary file 1 [file sensors-23-08332-s001.zip › sensors-2637596-supplementary.pdf]

Supplemental data

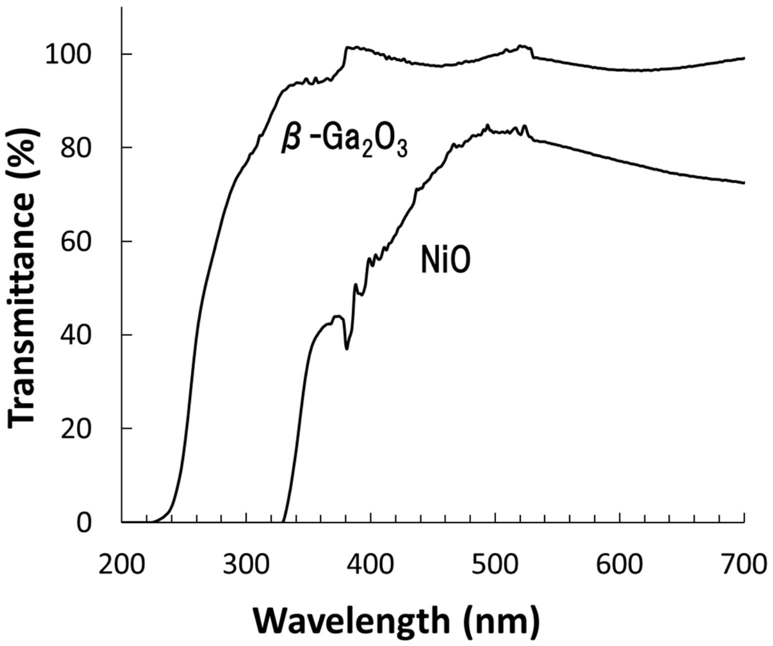

Figure S1. Optical transmission spectra of Li-doped NiO and β-Ga_2O_3 films formed on sapphire substrate.
